# Associations of kidney tests at medical facilities and health checkups with incidence of end-stage kidney disease: a retrospective cohort study

**DOI:** 10.1038/s41598-021-99971-w

**Published:** 2021-10-26

**Authors:** Ryuichi Yoshimura, Ryohei Yamamoto, Maki Shinzawa, Rie Kataoka, Mina Ahn, Nami Ikeguchi, Natsuki Wakida, Hiroshi Toki, Toshiki Moriyama

**Affiliations:** 1 Health and Counseling Center, Osaka University, 1-17 Machikaneyamacho, Toyonaka, 560-0043 Japan; 2 Department of Nephrology, Osaka University Graduate School of Medicine, 2-2-B6 Yamadaoka, Suita, 565-0871 Japan; 3 Health Promotion and Regulation, Department of Health Promotion Medicine, Osaka University Graduate School of Medicine, 1-17 Machikaneyamacho, Toyonaka, 565-0043 Japan; 4 Health Promotion Division, Neyagawa City Public Health Center, 28-22 Ikedanishicho, Neyagawa, 572-8533 Japan

**Keywords:** Epidemiology, Population screening, End-stage renal disease

## Abstract

No study has assessed the association between no health checkup and end-stage kidney disease (ESKD). This retrospective cohort study, including 69,147 adults aged ≥ 40 years in Japan who were insured by the National Health Insurance and the Late-Stage Medical Care System for the Elderly, assessed the associations of kidney tests at medical facilities and health checkups with incident ESKD. The main exposure was the histories of kidney tests using dipstick urinalysis and/or serum creatinine measurement at medical facilities and checkups in the past year: “checkups,” “no kidney test (without checkup),” and “kidney tests (without checkup)” groups. During the median observational period of 5.0 years, ESKD was observed in 246 (0.8%) men and 124 (0.3%) women. The “no kidney test” group was associated with ESKD in men (adjusted subhazard ratio of “no kidney test” vs. “checkups”: 1.66 [95% confidence interval, 1.04–2.65], but not in women. Age-specific subgroup analyses identified the “no kidney test” group as a high-risk population of ESKD in elderly men (1.30 [0.70–2.41] and 2.72 [1.39–5.33] in men aged 40–74 and ≥ 75 years, respectively). Elderly men with no kidney test at medical facilities and no health checkup were at higher risk of ESKD.

## Introduction

The growing number of patients with end-stage kidney disease (ESKD) undergoing dialysis treatment is a public health problem with an enormous economic burden^1–3^. In Japan, the number of elderly dialysis patients is increasing, with the elderly aged ≥ 75 years^4^ accounting for 42.9% of incident dialysis patients in 2018^5^. One beneficial approach to reduce the risk of incident ESKD is to screen for chronic kidney disease (CKD)^6^. Besides patients with diabetes and hypertension^7,8^, the elderly are potential candidates for CKD screening^9,10^. The annual health checkup program for all adults aged ≥ 40 years, introduced by the Japanese Ministry of Health, Labour and Welfare (MHLW) in 2008, has played a pivotal role in the population-based screening for CKD^11^, along with cardiometabolic diseases^12^. Because a Japanese study suggested that CKD screening using dipstick urinalysis and/or serum creatinine measurement was a cost-effective approach to prevent progression to ESKD^13^, the MHLW has encouraged adults aged ≥ 40 years to undergo CKD screening through the annual health checkup program, which includes dipstick urinalysis as a mandatory item and serum creatinine measurement as an optional item^14^. However, the proportion of the population undergoing health checkups is low at about 50% and 30% in adults aged 40–74 and ≥ 75 years, respectively^15,16^.

Several cohort studies showed that no health checkup was associated with mortality^17,18^, whereas the renal prognosis of adults with no checkup remains to be elucidated. Among adults with no checkup, some undergo kidney tests using dipstick urinalysis and/or serum creatinine measurement at medical facilities, as suggested by a Japanese cross-sectional study reporting that 64% of adults aged ≥ 65 years with no checkup underwent opportunistic screening, equivalent to health checkups, at medical facilities^19^. Others are patients under treatment for CKD, who have regularly kidney tests at medical facilities. Misclassification of adults with opportunistic CKD screening at medical facilities and patients under treatment for CKD as adults with no checkup potentially leads to biased estimates of the association between no checkup and incident ESKD. Thus, the clinical impact of no screening for CKD on renal prognosis should be assessed by comparing the incidence of ESKD in adults with no kidney test at medical facilities and no checkup with the incidence in those with checkups.

The present retrospective cohort study, the Neyagawa Health checkups and Health care in Kokuho database (NHHK) study, of 69,147 adults aged ≥ 40 years, including 22,767 (32.9%) adults aged ≥ 75 years, aimed to assess the associations of kidney tests using dipstick urinalysis and/or serum creatinine measurement at medical facilities and health checkups with the incidence of ESKD in the general population. Additionally, we assessed their associations in the elderly, who had a low proportion of health checkups.

## Methods

### Data source

We conducted a retrospective cohort study using the National Health Insurance Database of Japan (KDB) developed by the All-Japan Federation of National Health Insurance Organizations in 2012. The National Health Insurance (NHI) covers adults aged < 75 years who are mainly self-employed, retirees, and their non-working dependents, whereas the Late-Stage Medical Care System for the Elderly covers adults aged ≥ 75 or 65–74 years with certain disabilities. The KDB consists of data on the beneficiaries covered by the two insurers and includes data on insurance eligibility, monthly medical claims, and annual health checkups. The insured period of each beneficiary was ascertained by the dates of acquisition and loss of insurance eligibility. Beneficiaries who changed their insurers from the NHI to the Late-Stage Medical Care System for the Elderly after reaching the age of 75 years or disability certification can be identified by matching the data from the two insurers. The monthly medical claims data, including standardized codes for electronic claims processing by the MHLW, provides information on prescriptions, medical procedures, and laboratory, physiological, and radiological examinations without results.

We obtained the KDB for the period between April 2012 and March 2018 from the municipal government of Neyagawa City, Osaka Prefecture, Japan. Neyagawa City had a population of 242,087, including 23,759 (9.8%) adults aged ≥ 75 years as of April 1, 2013^20^.

### Participants

**Figure 1:**
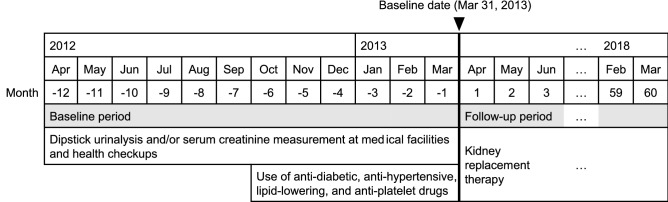
Measurements of the baseline and outcome variables.

**Figure 2:**
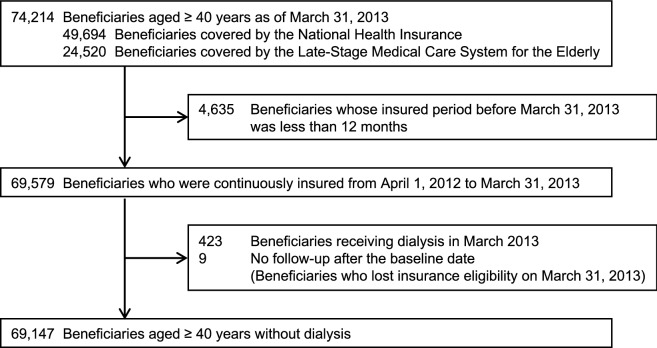
A flow diagram of study entry.

The baseline date was March 31, 2013. The study period was divided into a baseline period of 12 months from April 1, 2012, to March 31, 2013, and the follow-up period from April 1, 2013, to March 31, 2018 (Fig. 1). Eligible participants of the NHHK study were 74,214 beneficiaries aged ≥ 40 years at the baseline date (Fig. 2). During the study period, 9251 (12.5%) beneficiaries changed their insurers from the NHI to the Late-Stage Medical Care System for the Elderly. Of these beneficiaries, the insured period of 8991 (97.2%) beneficiaries was treated as continuous after the change in insurers. The end of the insured period of the remaining 260 (2.8%) beneficiaries was defined as the date of loss of eligibility for the NHI due to the lack of data on the Late-Stage Medical Care System for the Elderly. Of 69,579 (93.8%) beneficiaries who were continuously insured during the 1-year baseline period, we excluded 423 (0.6%) who were receiving dialysis and had medical claims codes corresponding to Japanese procedure codes for hemodialysis or peritoneal dialysis in March 2013 (Supplementary Table S1)^21^and nine (0.0%) beneficiaries without the follow-up period. We finally included 69,147 (93.2%) beneficiaries aged ≥ 40 years without dialysis.

The Ethics Committee of Health and Counseling Center, Osaka University approved the study protocol and waived informed consent due to the retrospective nature of the study (No. 2019-14). All procedures performed in studies involving human participants were in accordance with the ethical standards of the institutional and/or national research committee and with the 1964 Declaration of Helsinki and its later amendments or comparable ethical standards.

### Measurements

The baseline variables included age; sex; use of anti-diabetic, anti-hypertensive, lipid-lowering, and anti-platelet drugs; and smoking status (if beneficiaries underwent health checkups during the 1-year baseline period). Use of these drugs was determined based on the history of one or more prescriptions during the 6 months prior to the baseline date. The medical claims codes for drugs were converted to Anatomical Therapeutic Chemical (ATC) Classification System codes using the master data developed by the Japan Pharmaceutical Information Center (http://www.japic.or.jp) to identify anti-diabetic (ATC codes: A10)^22^, anti-hypertensive (C03A, C07, C08, and C09)^22^, lipid-lowering (C10AA, C10AX09, and C10BA)^23^, and anti-platelet drugs (B01AC) (Supplementary Table S2)^24^. Smoking status was classified into the absence and presence of current smoking using a health checkup questionnaire.

The main exposure was kidney tests at medical facilities and health checkups in the past year. We categorized beneficiaries into three groups: beneficiaries were divided into the “checkups” and “no checkup” groups using the history of health checkups during the 1-year baseline period, and the “no checkup” group was divided into two groups according to the absence and presence of kidney tests using dipstick urinalysis and/or serum creatinine measurement at medical facilities during the 1-year baseline period (the medical claims codes of 160000310 and 160019210, respectively^25^), namely “no kidney test” and “kidney tests” groups.

The outcome measure of the present study was the incidence of ESKD, defined as the initiation of kidney replacement therapy with hemodialysis, peritoneal dialysis, or kidney transplantation, using the Japanese procedure codes (Supplementary Table S1)^21,26^. The competing event was death before the incidence of ESKD, verified by the reason for the loss of insurance eligibility^27,28^. Because the monthly claims data did not include any information on the date, the observational period was designated as the number of months from the baseline date to (i) the incidence of ESKD, (ii) death, (iii) loss of insurance eligibility, or (iv) March 31, 2018, whichever came first (Fig. 1). If ESKD and death occurred in the same month, the incidence of ESKD was treated as the outcome event prior to death.

### Statistical analysis

To evaluate the associations of kidney tests at medical facilities and health checkups with the incidence of ESKD, the cumulative probabilities of the incidence of ESKD were estimated using the cause-specific cumulative incidence function (CIF) and compared using a weighted log-rank test, calculated using Stata’s s*tcrprep* command^29^. We performed competing risk regression analyses using Fine and Gray proportional subhazards models with death as a competing risk event^30^, whereby covariates from each prior model were retained as follows: Model 1 was unadjusted; Model 2 added age; Model 3 added use of anti-diabetic and anti-hypertensive drugs; Model 4 added use of lipid-lowering and anti-platelet drugs. The proportional subhazards assumption was checked using time interactions for all covariates. The effect modification of age with kidney tests and health checkups was assessed by incorporating their interaction term into Model 4. P for interaction < 0.10 was regarded as statistically significant. To clarify their interaction, we evaluated the associations of kidney tests and health checkups with the incidence of ESKD in adults aged 40–74 and ≥ 75 years.

To control for the imbalance of the baseline characteristics between the “checkups” and “no kidney test” groups, we performed a propensity score (PS) analysis. The PS was calculated using a multivariable-adjusted logistic regression model, which included baseline age and use of anti-diabetic, anti-hypertensive, lipid-lowering, and anti-platelet drugs as independent variables. The area under the receiver operating characteristic curve (AUC) was calculated to assess the predictive ability of the PS. After calculating the PS, each adult in the “no kidney test” group was matched to an adult in the “checkups” group with the closest PS at a ratio of 1:1 without replacement, using a standard greedy matching algorithm with a caliper width of 0.2 standard deviation of the logit of the PS^31^. The balance in baseline characteristics between the two groups after matching was examined using standardized differences. An absolute standardized difference (ASD) < 0.1 was regarded as balanced^32^. Among matched pairs, the cumulative probabilities of the incidence of ESKD were calculated using the cause-specific CIF and compared using a weighted log-rank test and an unadjusted Fine and Gray model.

To assess the association between no health checkup and incident ESKD, an additional analysis comparing the incidence of ESKD in the “no checkup” group with that in the “checkups” group was performed using Fine and Gray models with the aforementioned Models 1–4.

Continuous variables are expressed as median and interquartile range, and categorical variables are expressed as numbers and proportions. Statistical significance was set at P < 0.05, unless otherwise specified. Statistical analyses were performed using Stata (version 16.1; Stata Corp, http://www.stata.com) and R (version 4.0.3; The R Foundation for Statistical Computing, http://www.r-project.org).

## Results

### Baseline characteristics

Of 30,669 men, 9474 (30.9%), 9833 (32.1%), and 11,362 (37.0%) had checkups, no kidney test and no checkup, and kidney tests and no checkup at their baseline date, respectively. Of 38,478 women, 14,145 (36.8%), 10,311 (26.8%), and 14,022 (36.4%) were classified into the “checkups,” “no kidney test,” and “kidney tests” groups, respectively. Their baseline characteristics for men and women are listed in Table 1 separately. In both men and women, the “no kidney test” group had younger age and were less likely to use anti-diabetic, anti-hypertensive, lipid-lowering, and anti-platelet drugs compared with the “checkups” and “kidney tests” groups.

**Table 1:** Baseline characteristics stratified by kidney tests at medical facilities and health checkups in 30,669 men and 38,478 women.

		Checkups	No checkup	
			No kidney test	Kidney tests^b^
Men	Number	9474	9833	11,362
	Age, years	71 (65–76)	62 (48–70)	72 (65–78)
	Use of anti-diabetic drugs, n (%)	1222 (12.9)	105 (1.1)	2725 (24.0)
	anti-hypertensive drugs	4299 (45.4)	835 (8.5)	6566 (57.8)
	lipid-lowering drugs	1950 (20.6)	203 (2.1)	3009 (26.5)
	anti-platelet drugs	1677 (17.7)	284 (2.9)	3252 (28.6)
	Kidney tests at checkups^a^, n (%)	9456 (99.8)	0 (0.0)	0 (0.0)
	at medical facilities^b^	5873 (62.0)	0 (0.0)	11,362 (100.0)
	Current smokers, n (%)	2302 (24.3)	NA	NA
Women	Number	14,145	10,311	14,022
	Age, years	70 (65–76)	65 (55–76)	74 (66–81)
	Use of anti-diabetic drugs, n (%)	975 (6.9)	110 (1.1)	2,385 (17.0)
	anti-hypertensive drugs	5702 (40.3)	1098 (10.6)	7818 (55.8)
	lipid-lowering drugs	4520 (32.0)	451 (4.4)	5143 (36.7)
	anti-platelet drugs	1924 (13.6)	339 (3.3)	3246 (23.1)
	Kidney tests at checkups^a^, n (%)	14,127 (99.9)	0 (0.0)	0 (0.0)
	at medical facilities^b^	8360 (59.1)	0 (0.0)	14,022 (100.0)
	Current smokers, n (%)	886 (6.3)	NA	NA

Data are presented as median (25–75%) or n (%).

NA, not available.

^a^Dipstick urinalysis and/or serum creatinine measurement at health checkups in the past year.

^b^Dipstick urinalysis and/or serum creatinine measurement at medical facilities in the past year.

### Kidney tests at medical facilities, health checkups, and the incidence of ESKD

**Figure 3:**
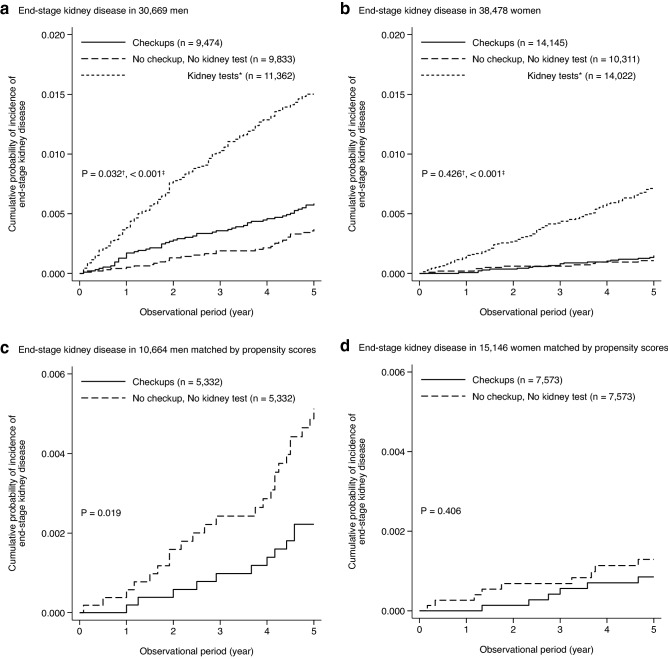
Cumulative probabilities of the incidence of end-stage kidney disease in 30,669 men (**a**), 38,478 women (**b**), and 5332 male (**c**) and 7573 female pairs (**d**) matched by propensity scores. *Dipstick urinalysis and/or serum creatinine measurement at medical facilities in the past year. ^†^P for “checkups” vs. “no kidney test” groups. ^‡^P for “checkups” vs. “kidney tests” groups.

During the median observational period of 5.0 years (interquartile range, 5.0–5.0), ESKD was observed in 246 (0.8%) men and 124 (0.3%) women, but none of them underwent kidney transplantation. The cumulative probability of the incidence of ESKD was significantly lower in the “no kidney test” group than in the “checkups” group among men (P = 0.032 and 0.426 for men and women, respectively), whereas the “kidney tests” group had a higher cumulative probability of incident ESKD than the “checkups” group among both men and women (P < 0.001 for both men and women) (Fig. 3a,b). The unadjusted Fine and Gray model showed that the “no kidney test” and “kidney tests” groups were significantly associated with a lower and a higher incidence of ESKD in men, respectively (subhazard ratios [SHR] of “checkups,” “no kidney test,” and “kidney tests”: 1.00 [reference], 0.61 [95% confidence interval, 0.39–0.95], and 2.60 [1.90–3.54], respectively) (Table 2). After adjusting for use of anti-hypertensive and anti-diabetic drugs, the “no kidney test” group was found to be at a significantly higher risk of ESKD (SHR of “no kidney test” vs. “checkups”: 1.66 [1.04–2.65] in model 4). The “kidney tests” group was also significantly associated with the higher incidence of ESKD even after adjusting for clinically relevant factors (SHR of “kidney tests” vs. “checkups”: 1.87 [1.35–2.58] in model 4). In contrast to men, the multivariable-adjusted model showed that in women, the “no kidney test” group was not significantly associated with the incidence of ESKD, whereas the “kidney tests” group was (Table 2).

**Table 2:** Associations of kidney tests at medical facilities and health checkups with the incidence of end-stage kidney disease.

		Checkups	No checkup	
			No kidney test	Kidney tests^a^
Men	Number	9474	9833	11,362
	Observational period, year	5.0 (5.0–5.0)	5.0 (4.1–5.0)	5.0 (3.8–5.0)
	Incidence of ESKD, n (%)	53 (0.6)	31 (0.3)	162 (1.4)
	IR per 1000 PY (95% CI)	1.2 (0.9–1.6)	0.7 (0.5–1.1)	3.4 (2.9–4.0)
	Model 1 SHR (95% CI)	1.00 (reference)	0.61 (0.39–0.95)	2.60 (1.90–3.54)
	Model 2 SHR (95% CI)	1.00 (reference)	0.78 (0.49–1.23)	2.46 (1.80–3.37)
	Model 3 SHR (95% CI)	1.00 (reference)	1.63 (1.02–2.60)	1.92 (1.39–2.65)
	Model 4 SHR (95% CI)	1.00 (reference)	1.66 (1.04–2.65)	1.87 (1.35–2.58)
	P for interaction^b^	–	0.014	0.195
Women	Number	14,145	10,311	14,022
	Observational period, year	5.0 (5.0–5.0)	5.0 (4.9–5.0)	5.0 (4.6–5.0)
	Incidence of ESKD, n (%)	20 (0.1)	10 (0.1)	94 (0.7)
	IR per 1000 PY (95% CI)	0.3 (0.2–0.5)	0.2 (0.1–0.4)	1.6 (1.3–1.9)
	Model 1 SHR (95% CI)	1.00 (reference)	0.73 (0.34–1.56)	4.82 (2.97–7.80)
	Model 2 SHR (95% CI)	1.00 (reference)	0.78 (0.37–1.65)	4.49 (2.73–7.37)
	Model 3 SHR (95% CI)	1.00 (reference)	1.53 (0.72–3.25)	3.18 (1.93–5.24)
	Model 4 SHR (95% CI)	1.00 (reference)	1.51 (0.70–3.24)	3.15 (1.91–5.19)
	P for interaction^b^	–	0.131	0.120

Model 1, unadjusted.

Model 2, adjusted for age (years).

Model 3, adjusted for covariates in model 2 and use of anti-diabetic and anti-hypertensive drugs.

Model 4, adjusted for covariates in model 3 and use of lipid-lowering and anti-platelet drugs.

CI, confidence interval; ESKD, end-stage kidney disease; IR, incidence rate; PY, person-years; SHR, subhazard ratio.

^a^Dipstick urinalysis and/or serum creatinine measurement at medical facilities in the past year.

^b^P for interaction of age with kidney tests at medical facilities and health checkups.

### Subgroup analyses stratified by age

**Figure 4:**
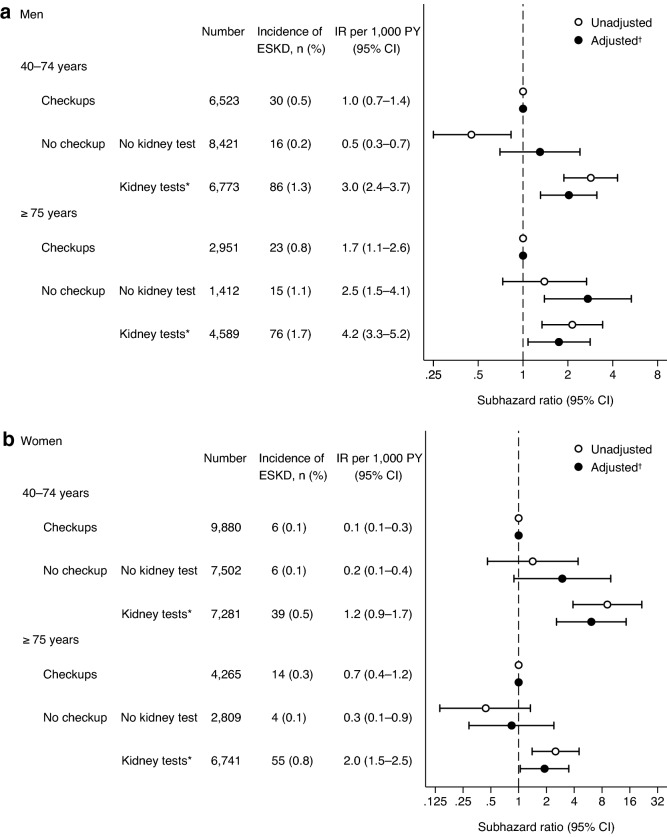
Associations of kidney tests at medical facilities and health checkups with the incidence of end-stage kidney disease stratified by age. CI, confidence interval; ESKD, end-stage kidney disease; IR, incidence rate; PY, person-years. *Dipstick urinalysis and/or serum creatinine measurement at medical facilities in the past year. ^†^Adjusted for age (years) and use of anti-diabetic, anti-hypertensive, lipid-lowering, and anti-platelet drugs.

Because of a significant interaction of age with kidney tests and health checkups in men (P for interaction of “no kidney test” and “kidney tests” groups: 0.014 and 0.195, respectively), we assessed the associations of kidney tests and health checkups with the incidence of ESKD in men aged 40–74 and ≥ 75 years, separately. The “no kidney test” group was associated with the incidence of ESKD in men aged ≥ 75 years, but not in those aged 40–74 years (adjusted SHR of “no kidney test” vs. “checkups”: 1.30 [0.70–2.41] and 2.72 [1.39–5.33] in men aged 40–74 and ≥ 75 years, respectively) (Fig. 4a). In contrast to men, no significant interaction was observed in women (P for interaction of “no kidney test” and “kidney tests” groups: 0.131 and 0.120, respectively). The “no kidney test” group was not associated with the incidence of ESKD in either women aged 40–74 years or ≥ 75 years (adjusted SHR of “no kidney test” vs. “checkups”: 2.97 [0.89–9.94] and 0.84 [0.29–2.41] in women aged 40–74 and ≥ 75 years, respectively) (Fig. 4b).

### Sensitive analyses using PS matching

A PS analysis confirmed the robustness of the results comparing the incidence of ESKD in the “no kidney test” group with that in the “checkups” group. To control for the imbalance of the baseline characteristics between the “checkups” and “no kidney test” groups, we calculated the PS using the logistic regression model with adults in the “no kidney test” group as a dependent variable. The model had moderate discrimination in determining adults with no kidney test and no checkup (AUC = 0.805 and 0.748 in men and women, respectively). Each adult in the “no kidney test” group was matched to an adult in the “checkups” group, resulting in 5332 male and 7573 female pairs. The baseline characteristics between the two groups were well balanced, with all ASD less than 0.1 (Table 3). The cumulative probability of the incidence of ESKD was significantly higher in the “no kidney test” group than in the “checkups” group among male pairs, but not among female pairs (Fig. 3c,d). The unadjusted Fine and Gray model showed that the “no kidney test” group was at a higher risk of ESKD compared with the “checkups” group in men, but not in women (Table 4).

**Table 3:** Baseline characteristics of 5332 male and 7573 female pairs matched by propensity scores.

		Checkups	No kidney test and no checkup^a^	ASD
Men	Number	5332	5332	
	Age, years	69 (62–74)	68 (62–74)	0.019
	Use of anti-diabetic drugs, n (%)	111 (2.1)	105 (2.0)	0.008
	anti-hypertensive drugs	858 (16.1)	828 (15.5)	0.015
	lipid-lowering drugs	275 (5.2)	203 (3.8)	0.065
	anti-platelet drugs	395 (7.4)	278 (5.2)	0.090
Women	Number	7573	7573	
	Age, years	68 (63–74)	68 (60–77)	< 0.001
	Use of anti-diabetic drugs, n (%)	158 (2.1)	110 (1.5)	0.048
	anti-hypertensive drugs	1,092 (14.4)	1,098 (14.5)	0.002
	lipid-lowering drugs	436 (5.8)	451 (6.0)	0.008
	anti-platelet drugs	416 (5.5)	338 (4.5)	0.047

Data are presented as median (25–75%) or n (%).

ASD, absolute standardized difference.

^a^No dipstick urinalysis and no serum creatinine measurement at medical facilities and no health checkup in the past year.

**Table 4:** Associations of kidney tests at medical facilities and health checkups with the incidence of end-stage kidney disease among propensity score-matched pairs.

		Checkups	No kidney test and no checkup^a^
Men	Number	5332	5332
	Observational period, year	5.0 (5.0–5.0)	5.0 (4.5–5.0)
	Incidence of ESKD, n (%)	11 (0.2)	24 (0.5)
	IR per 1000 PY (95% CI)	0.5 (0.3–0.8)	1.1 (0.7–1.6)
	Unadjusted SHR (95% CI)	1.00 (reference)	2.30 (1.13–4.70)
Women	Number	7573	7573
	Observational period, year	5.0 (5.0–5.0)	5.0 (5.0–5.0)
	Incidence of ESKD, n (%)	6 (0.1)	9 (0.1)
	IR per 1000 PY (95% CI)	0.2 (0.1–0.4)	0.3 (0.1–0.5)
	Unadjusted SHR (95% CI)	1.00 (reference)	1.54 (0.55–4.33)

CI, confidence interval; ESKD, end-stage kidney disease; IR, incidence rate; PY, person-years; SHR, subhazard ratio.

^a^No dipstick urinalysis and no serum creatinine measurement at medical facilities and no health checkup in the past year.

### No health checkup and the incidence of ESKD

Regarding the association between undergoing no health checkup and the incidence of ESKD, unadjusted and adjusted Fine and Gray models showed that the “no checkup” group was significantly associated with the incidence of ESKD compared with the “checkups” group in both men and women. (Supplementary Table S3).

## Discussion

This retrospective cohort study, which included 69,147 adults aged ≥ 40 years, revealed that men with no kidney test using dipstick urinalysis and/or serum creatinine measurement at medical facilities and no health checkup were at a significantly higher risk of ESKD than those with checkups, especially in the elderly aged ≥ 75 years. These results suggest that elderly men with no kidney test and no checkup were a potential target for CKD screening to prevent ESKD in the general population. The advantages of this study were a larger sample size, which included 22,767 (32.9%) elderly adults aged ≥ 75 years, and the comparison of the incidence of ESKD in adults with no kidney test and no checkup with that in those with checkups.

Although no study has reported the association between no health checkup and the incidence of ESKD, a Japanese cohort study suggested that no health checkup was associated with mortality. The Osaki NHI Cohort Study, which included 48,775 adults aged 40–79 years, showed that adults with no checkup were at a significantly higher risk of all-cause mortality than those with checkups^17^. Similar to the previous study, the present study showed that undergoing no checkup was significantly associated with the incidence of ESKD. Additionally, the present study clarified that adults with no kidney test and no checkup were at a higher risk of ESKD in elderly men, but not in young men, whereas the previous study did not assess an age-dependent association between undergoing no checkup and mortality. The findings of the present study suggest that municipal healthcare planning should include the promotion of CKD screening in elderly men to prevent ESKD.

One of the potential candidates for the age-dependent association of undergoing no kidney test and no checkup with the incidence of ESKD in men might be smoking status. Because several studies showed that current smokers were less likely to undergo checkups^16,33^, men with no kidney test and no checkup probably had a higher prevalence of current smokers than those with checkups in the present study. Regarding the association between current smokers and renal prognosis, recent cohort studies showed that current smokers had a faster decline in estimated glomerular filtration rate (eGFR)^34^ and a higher risk of ESKD in elderly adults than in young adults^35^. Accordingly, in the present study, adults with no kidney test and no checkup, who probably had a higher prevalence of current smokers, were at a higher risk of ESKD, especially in elderly men.

Among adults with no checkup, those with kidney tests had a higher risk of ESKD than those with checkups, which was probably attributed to a difference in the prevalence of CKD between adults with kidney tests and those with checkups. Although baseline kidney function, including eGFR and urinary protein, was not available for adults with no checkup in the present study, adults with kidney tests had a higher prevalence of drug use for cardiometabolic diseases than those with checkups. Because patients with cardiometabolic diseases are high-risk populations for CKD^36,37^, the prevalence of CKD was likely to be higher in adults with kidney tests than in those with checkups. In contrast to adults with kidney tests, those with no kidney test had a lower prevalence of drug use for cardiometabolic diseases than those with checkups, suggesting that the prevalence of CKD in adults with no kidney test was probably lower than that in those with checkups. If we could control for the prevalence of CKD, the risk of ESKD in adults with kidney tests would be attenuated compared with the risk observed in the present study, whereas the risk in adults with no kidney test would be enhanced. Although the results of this study suggest that health checkups might be effective in preventing ESKD, the efficacy should be evaluated in well-designed randomized controlled trials.

The incidence of ESKD was higher in men than in women in the present study. One possible reason for this gender difference in the incidence of ESKD was a difference in the prevalence of cardiometabolic diseases between men and women. Several previous studies showed that besides CKD^38^, cardiometabolic diseases, including diabetes^39^ and cardiovascular diseases^40^, were crucial risk factors for ESKD. In the present study, among 23,468 adults with checkups, the prevalence of a positive result of proteinuria, use of anti-diabetic drugs, and a past history of cardiovascular diseases was higher in men than in women (Supplementary Table S4). Although unadjusted and adjusted Fine and Gray models in adults with checkups showed that men were associated with the incidence of ESKD, an adjustment for urinary protein, use of anti-diabetic drugs, and a past history of cardiovascular diseases attenuated the association between men and the incidence of ESKD at the level of Model 3 in Supplementary Table S5 (SHR of men [vs. women] adjusted for urinary protein, use of anti-diabetic drugs, and a past history of cardiovascular diseases: 2.39 [1.41–4.03] vs. 2.29 [1.24–4.23] in model 3 in Supplementary Table S5), strongly suggesting that the higher prevalence of a positive result of proteinuria, use of anti-diabetic drugs, and a past history of cardiovascular diseases was one of the major confounding factors for the high risk of ESKD in men. However, even after adjusting for these potential confounding factors, the risk of ESKD in men was significantly high, implying that unmeasured confounding factors even contributed to this gender difference.

The present study has several limitations. First, the small number of incidences of ESKD in women hindered meaningful statistical analysis. A large cohort of women is essential to evaluate the associations of kidney tests at medical facilities and health checkups with incident ESKD. Second, the generalizability of the findings of the present study should be examined in different cohorts, given the differences in national health checkup programs among countries. Compared with Japan, several countries have health checkup programs for a more narrowly targeted population and provide health checkups less frequently^41,42^. In England, the National Health Service Health Check is conducted for adults aged 40–74 years without cardiometabolic diseases or cardiovascular diseases every 5 years^41^. Third, the present study had no information on the past history of kidney transplantation at the baseline date. In Japan, only 2% of patients with ESKD underwent kidney transplantation in 2018^43^. Assuming that the prevalence of a past history of kidney transplantation was very low in the present study, the small number of adults with kidney transplantation was unlikely to lead to biased results. Fourth, the present study assessed the 1-year checkup history and the incidence of ESKD. Some people might undergo health checkups every two years, three years, or more. A clinical impact of the checkup history during the 2-year, 3-year, or longer period on the incidence of ESKD could not be assessed in the present study because of a limited study period and number of participants. A further study with a longer study period is necessary to assess an association between the longer checkup history and the incidence of ESKD. Fifth, the associations of kidney tests and checkups with the incidence of ESKD may be confounded by unmeasured lifestyle factors. The previous studies showed that adults with no checkup were less likely to exercise and eat fruits and vegetables^33,44^. Additionally, a cross-sectional study showed that adults who did not visit general practitioners were prone to have unhealthy lifestyle factors, including physical inactivity and low fruit and vegetable intake^45^. In the present study, the proportion of medical facility visits, which was defined using the Japanese procedure codes (A000, A001, and A002), was 92.1%, 44.9% and 99.1% in those with checkups, those with no kidney test and no checkup, and those with kidney tests and no checkup, respectively. Therefore, adults with no kidney test and no checkup possibly had a higher prevalence of physical inactivity and low fruit and vegetable intake than those with checkups and those with kidney tests and no checkup. Because several cohort studies showed that physical inactivity^46^ and low fruit and vegetable intake^47^ were associated with a higher risk of ESKD, the associations of kidney tests and checkups with incident ESKD were confounded by these lifestyle factors. Sixth, health guidance after checkups might affect the associations of kidney tests and checkups with incident ESKD. In FY2012, 2.9% of adults aged 40–74 years who underwent checkups received specific health guidance targeting metabolic syndrome in Japan^48^. Given that the prevalence of the health guidance in Neyagawa City was at the same level as that in Japan, the small number of adults with the health guidance was unlikely to affect the results of this study. Further studies are essential to assess the association between health guidance and incident ESKD.

In conclusion, the present study identified the elderly with no kidney test at medical facilities and no health checkup as a high-risk population of ESKD in men, but not in women. These results suggest that elderly men with no kidney test at medical facilities and no health checkup are potential candidates for CKD screening and should be encouraged to undergo health checkups in order to prevent the incidence of ESKD.

## Supplementary Information

Supplementary Information.

## Data Availability

The data presented in this study cannot be shared. These data originate from the municipal government of Neyagawa City and are not publicly available. Restrictions apply to the availability of these data, which used under license and ethical approval.
