# Supplementary material for: Associations of kidney tests at medical facilities and health checkups with incidence of end-stage kidney disease: a retrospective cohort study
Source: Sci Rep. 2021 Oct 26;11:20717. doi: 10.1038/s41598-021-99971-w (PMC8548563; doi:10.1038/s41598-021-99971-w)
Supplement: Supplementary file 1 — Supplementary Information. [file 41598_2021_99971_MOESM1_ESM.pdf]

Associations of kidney tests at medical facilities and health checkups with incidence of end-stage kidney disease: a retrospective cohort study

Ryuichi Yoshimura,[1] Ryohei Yamamoto*,[1–3] Maki Shinzawa,[2] Rie Kataoka,[4] Mina Ahn,[4] Nami Ikeguchi,[4] Natsuki Wakida,[4] Hiroshi Toki,[1] and Toshiki Moriyama[1–3]

[1]Health and Counseling Center, Osaka University

[2]Department of Nephrology, Osaka University Graduate School of Medicine

[3]Health Promotion and Regulation, Department of Health Promotion Medicine, Osaka University Graduate School of Medicine

[4]Health Promotion Division, Neyagawa City Public Health Center

Supplementary Table S1. Definitions of end-stage kidney disease using medical claims codes corresponding to Japanese procedure codes

	Procedure codes	Medical claims codes
Hemodialysis	J038*	140007710, 140007910, 140008170, 140033770, 140036710, 140051010, 140051110, 140052570, 140052810, 140052970, 140055970
	C102-2	114009310, 114009410
	C156	114009510
Peritoneal dialysis	J042	140008510, 140008770, 140008810, 140054850, 140054950
	C102	114003510, 114003610
	C154	114006510, 114008250
	C155	114006610
Kidney transplantation	K780	150196310, 150196570
	K780-2	150196410, 150338610
	K709-5	150324810

*Excluding J038-2 for continuous hemodiafiltration

Supplementary Table S2. Definitions of drugs using Anatomical Therapeutic Chemical (ATC) Classification System codes

	ATC code	Drug name
Anti-diabetic drugs	A10AB	insulin (human), insulin lispro, insulin aspart, insulin glulisine
	A10AC	insulin (human), insulin lispro
	A10AD	insulin (human), insulin lispro, insulin aspart, insulin degludec and insulin aspart
	A10AE	insulin glargine, insulin detemir, insulin degludec, protamine zinc insulin
	A10BA	metformin, buformin
	A10BB	glibenclamide, chlorpropamide, tolbutamide, gliclazide, glimepiride, acetohexamide, glyclopyramid
	A10BD	metformin and pioglitazone, glimepiride and pioglitazone, metformin and vildagliptin, pioglitazone and alogliptin, metformin and alogliptin, mitiglinide and voglibose, teneligliptin and canagliflozin
	A10BF	acarbose, miglitol, voglibose
	A10BG	pioglitazone
	A10BH	sitagliptin, vildagliptin, saxagliptin, alogliptin, linagliptin, teneligliptin, anagliptin, trelagliptin, omarigliptin
	A10BJ	exenatide, liraglutide, lixisenatide, dulaglutide
	A10BK	dapagliflozin, canagliflozin, empagliflozin, ipragliflozin, luseogliflozin, tofogliflozin
	A10BX	repaglinide, nateglinide, mitiglinide
	A10XA	epalrestat
Anti-hypertensive drugs	C03AA	hydrochlorothiazide, trichlormethiazide, benzylhydrochlorothiazide
	C07AA	alprenolol, oxprenolol, pindolol, propranolol, sotalol, nadolol, carteolol, bufetolol, arotinolol, nipradilol, tilisolol
	C07AB	metoprolol, atenolol, acebutolol, betaxolol, bevantolol, bisoprolol, celiprolol, esmolol, landiolol
	C07AG	labetalol, carvedilol, arotinolol, amosulalol
	C08CA	amlodipine, felodipine, nicardipine, nifedipine, nisoldipine, nitrendipine, nilvadipine, manidipine, barnidipine, cilnidipine, benidipine, efonidipine, azelnidipine
	C08DA	verapamil

	C08DB	diltiazem
	C08EA	bepridil
	C08EX	aranidipine
	C09AA	captopril, enalapril, lisinopril, perindopril, quinapril, benazepril, cilazapril, trandolapril, delapril, temocapril, imidapril, alacepril
	C09CA	losartan, valsartan, irbesartan, candesartan, telmisartan, olmesartan medoxomil, azilsartan medoxomil
	C09DA	losartan and diuretics, valsartan and diuretics, irbesartan and diuretics, candesartan and diuretics, telmisartan and diuretics
	C09DB	valsartan and amlodipine, telmisartan and amlodipine, irbesartan and amlodipine, candesartan and amlodipine, olmesartan medoxomil and azelnidipine, valsartan and cilnidipine, azilsartan and amlodipine
	C09DX	telmisartan, amlodipine, and hydrochlorothiazide
	C09XA	aliskiren
Lipid-lowering drugs	C10AA	simvastatin, pravastatin, fluvastatin, atorvastatin, rosuvastatin, pitavastatin
	C10AX09	ezetimibe
	C10BA	NA
Anti-platelet drugs	B01AC	clopidogrel, ticlopidine, acetylsalicylic acid, dipyridamole, epoprostenol, iloprost, beraprost, treprostinil, prasugrel, cilostazol, ticagrelor, selexipag, acetylsalicylic acid, combinations with proton pump inhibitors, limaprost alfadex, ethyl icosapentate, sarpogrelate, ozagrel

NA, not applicable

Supplementary Table S3. An association between no health checkup and the incidence of end-stage kidney disease

		Checkups	No checkup
Men	Number	9,474	21,195
	Observational period, year	5.0 (5.0–5.0)	5.0 (3.9–5.0)
	Incidence of ESKD, n (%)	53 (0.6)	193 (0.9)
	IR per 1000 PY (95% CI)	1.2 (0.9–1.6)	2.2 (1.9–2.5)
	Model 1 SHR (95% CI)	1.00 (Reference)	1.71 (1.26–2.31)
	Model 2 SHR (95% CI)	1.00 (Reference)	1.84 (1.36–2.50)
	Model 3 SHR (95% CI)	1.00 (Reference)	1.87 (1.37–2.53)
	Model 4 SHR (95% CI)	1.00 (Reference)	1.83 (1.34–2.49)
Women	Number	14,145	24,333
	Observational period, year	5.0 (5.0–5.0)	5.0 (4.8–5.0)
	Incidence of ESKD, n (%)	20 (0.1)	104 (0.4)
	IR per 1000 PY (95% CI)	0.3 (0.2–0.5)	1.0 (0.8–1.2)
	Model 1 SHR (95% CI)	1.00 (Reference)	3.13 (1.94–5.05)
	Model 2 SHR (95% CI)	1.00 (Reference)	3.02 (1.86–4.89)
	Model 3 SHR (95% CI)	1.00 (Reference)	2.84 (1.74–4.61)
	Model 4 SHR (95% CI)	1.00 (Reference)	2.81 (1.72–4.58)

CI, confidence interval; ESKD, end-stage kidney disease; IR, incidence rate; PY, person-years; SHR, subhazard ratio

Model 1, unadjusted

Model 2, adjusted for age (years)

Model 3, adjusted for covariates in model 2 and use of anti-diabetic and anti-hypertensive drugs

Model 4, adjusted for covariates in model 3 and use of lipid-lowering and anti-platelet drugs

Supplementary Table S4. Baseline characteristics of 14,064 women and 9,404 men with health checkups

	Women	Men
Number	14,064	9,404
Age, year	70 [65–76]	71 [65–76]
Current smokers, n (%)	883 (6.3)	2,286 (24.3)
Drinking frequency, Rare, n (%)	10,048 (71.4)	3,365 (35.8)
Occasional	2,682 (19.1)	1,707 (18.2)
Daily	1,334 (9.5)	4,332 (46.1)
Body mass index, kg/m^2	22.7 ± 3.5	23.5 ± 3.2
Systolic blood pressure, mmHg	131 ± 17	134 ± 17
Diastolic blood pressure, mmHg	75 ± 13	78 ± 11
Low-density lipoprotein cholesterol, mg/dL	128 ± 31	119 ± 32
Hemoglobin A1c, %	5.4 ± 0.7	5.5 ± 0.8
Urinary protein, negative or trace, n (%)	13,144 (93.5)	8,314 (88.4)
1+ or more	920 (6.5)	1,090 (11.6)
Use of anti-hypertensive drugs, n (%)	5,661 (40.3)	4,270 (45.4)
lipid-lowering drugs	4,493 (32.0)	1,937 (20.6)
anti-diabetic drugs	968 (6.9)	1,212 (12.9)
Past history of cardiovascular disease, n (%)	1,756 (12.5)	1,593 (16.9)
kidney disease	73 (0.5)	98 (1.0)

Data are presented as mean ± standard deviation, median (25%–75%), or n (%)

Supplementary Table S5. An association between gender and the incidence of ESKD in adults with health checkups

	Women	Men
Number	14,064	9,404
Observational period, year	5.0 (5.0–5.0)	5.0 (5.0–5.0)
Incidence of ESKD, n (%)	20 (0.1)	53 (0.6)
IR per 1,000 PY (95% CI)	0.3 (0.2–0.5)	1.2 (0.9–1.6)
Model 1 SHR (95% CI)	1.00 (Reference)	3.96 (2.37–6.63)
Model 2 SHR (95% CI)	1.00 (Reference)	3.51 (1.96–6.29)
Model 3 SHR (95% CI)	1.00 (Reference)	2.29 (1.24–4.23)

CI, confidence interval; ESKD, end-stage kidney disease; SHR, subhazard ratio

Model 1, unadjusted

Model 2, adjusted for age (years), current smokers, drinking frequency (rare, occasional, and daily), body mass index (kg/m^2), systolic blood pressure (mmHg), low-density lipoprotein cholesterol (mg/dL), hemoglobin A1c (%), use of anti-hypertensive and lipid-lowering drugs, and a past history of kidney disease

Model 3, adjusted for covariates in model 2 and urinary protein (negative or trace and 1+ or more), use of anti-diabetic drugs, and a past history of cardiovascular disease
